# Impact of BMI on Long-Term Outcomes in Patients with ST-Segment Elevation Myocardial Infarction after Primary Percutaneous Coronary Intervention

**DOI:** 10.1155/2022/6210204

**Published:** 2022-01-31

**Authors:** Jinwen Wang, Changhua Wang, Zhechun Zeng, Huijuan Zuo

**Affiliations:** ^1^Beijing Anzhen Hospital, Capital Medical University, Beijing Institute of Heart Lung and Blood Vessel Disease, Beijing 100029, China; ^2^Department of Cardiology, Beijing An Zhen Hospital, Capital Medical University, Beijing 100029, China

## Abstract

**Aim:**

Obesity paradox remains a point of debate in ST-segment elevation myocardial infarction (STEMI) patients. The aim of this study was to examine the relationship between body mass index (BMI) and clinical outcomes in STEMI patients undergoing primary percutaneous coronary intervention (PPCI).

**Methods:**

Outcomes were assessed in 1429 STEMI patients undergoing PPCI between January 2009 and January 2010 in Beijing. Patients were classified into 6 groups according to age (the younger and elderly groups consisting of patients ≤65 and > 65 years old) and baseline BMI (normal weight, BMI < 24 kg/m^2^; overweight, 24 kg/m^2^ ≤BMI < 28 kg/m^2^; obese, BMI ≥ 28 kg/m^2^). The primary outcome was death, acute myocardial infarction (AMI), or revascularization.

**Results:**

On long-term follow-up (mean follow-up of 59 months), 13.9% of patients experienced the adverse event. Multivariate logistic regression analyses showed that low BMI was a significant predictor of the primary outcome only in the younger group. The odds ratio for overweight in comparison with normal weight was 0.741 (95% CI: 0.413–0.979; *p* = 0.038), the odds radio for obesity in comparison with normal-weight patients was 0.508 (95% CI: 0.344–0.750; *p* = 0.016) in the younger group. In the elderly group, diabetes, hypertension, triple disease, regular exercise, angiotensin-converting enzyme inhibitor (ACEI) or angiotensin II receptor blockers (ARBs) use after discharge, and bleeding complication were associated with primary outcome.

**Conclusion:**

The obesity paradox was recognized only in the younger age group in STEMI patients undergoing PPCI.

## 1. Introduction

Obesity is highly prevalent among Chinese adults, and it has become a major public health challenge in China [1, 2]. Obesity increases a number of cardiovascular disease (CVD) risk factors, such as hypertension, dyslipidemia, and diabetes mellitus (DM), and is associated with the incidence of cardiovascular diseases and mortality [3, 4]. However, some controversial studies have demonstrated that overweight or obese patients with CVD may have a better prognosis than underweight or normal-weight CVD patients [5–7]. This phenomenon has been recognized as the “obesity paradox.” This “obesity paradox” has also been reported in patients with ST-segment elevation myocardial infarction (STEMI) who underwent primary percutaneous coronary intervention (PPCI). These studies showed that obesity/overweight patients had a better prognosis, with a lower mortality and recurrent acute myocardial infarction (AMI) incidence than normal-weight patients.

As expected, “obesity paradox” remains a point of debate. In several cases, methodological biases and the presence of confounders may account for these relationships. For example, age, smoking status, and chronic disease may be important confounders [8–11]. The obesity paradox may be due to the fact that patients with high BMI were younger than patients with low BMI. Previous reports showed that smoking and chronic diseases are associated with high mortality and low BMI values, which may partly account for the obesity paradox. In addition, it has been suggested that obese patients tend to be treated more aggressively and have less bleeding complications than their leaner counterparts [12, 13]. Therefore, the aim of the present study was to evaluate the relationship between BMI and clinical outcomes in patients with STEMI undergoing PPCI after adjusting for multiple confounders, including age, smoking, chronic disease, treatment after discharge, and bleeding complications.

## 2. Materials and Methods

### 2.1. Study Design and Population

#### 2.1.1. Sample Size

The *Z*-test with unpooled variance was used to calculate the sample size. The required sample size was estimated based on similar cohort studies, which reported long-term MACE (major adverse cardiac events) prevalence of 15.0% in normal patients and 9-10% in obesity or overweight patients, respectively [14]. Allowing a power of 80% and *⍺* error of 1.67%, we arrived at a sample size of 1323. Considering the dropout rate of 10%, a sample size of 1450 cases is enough. The sample size was estimated using PASS statistical software package.

### 2.2. Study Population

We consecutively recruited patients who aged >18 years and had an STEMI of <24-hour duration from the onset of symptoms until arrival at the Department of Cardiology in the medical institution in Beijing between January 2009 and January 2010. Patients were treated according to the current guidelines for the STEMI management. PPCI was performed by 1 of 4 operators, using standard techniques. Those who had one or more of the following factors were excluded from this study: (1) patients undergoing coronary artery bypass grafting (CABG); (2) patients with mechanical complications (ventricular free wall rupture, ventricular septal rupture, and papillary muscle rupture with severe mitral regurgitation); (3) patients undergoing thrombolysis; and (4) patients who experienced failure of stent implantation. All patients signed the written informed consent. The study was approved by the institutional review boards of the sixth clinical institute, Capital Medical University, Beijing, China.

### 2.3. Follow-Up

A follow-up visit and telephone interview, conducted by a medical doctor, were scheduled at 30 days, three months, six months, one year, and then yearly (maximum of 60 months of follow-up). We ascertained patients' persistence with prescribed guideline-directed medications after discharge, including angiotensin-converting enzyme inhibitor (ACEI) or angiotensin II receptor blockers (ARBs), aspirin, beta-blockers, and statins. Medication usage at follow-up was based on patients' self-report assessed via in-person or telephone interview. At each of these interviews, patients were asked to collect all their current medications and read each medicine to the interviewer. Persistence was assessed for use of aspirin, statins, *β*-blockers, and ACEI/ARBs individually.

At each interview, patients will be required to complete an interviewer-led questionnaire with questions on lifestyle information including (1) regular exercise, (2) smoking cessation, (3) reduced fried foods and meat, and (4) sleeping time. Patient's responses on smoking, diet, and exercise were based on the following Likert scale: never, rarely, sometimes, very often, and always. Those who answered “very often” or “always” for each question were considered to have a high adherence to each behavior.

### 2.4. Outcomes

The primary outcome was MACE. This was a composite of death from any cause, recurrent AMI, or the need for coronary revascularization. One of the researchers collected all-cause mortality, coronary revascularization, and recurrent AMI data during the follow-up by analyzing the information available in the medical records of the Medical Central of China.

### 2.5. Definition

STEMI was defined as chest pain suggestive of myocardial ischemia for at least 30 minutes within the previous 12 hours, accompanied by > 1 mm (0.1 mV) ST-segment elevation in ≥2 contiguous leads and later confirmed by creatine kinase (CK) and CK-MB increases and/or troponin increase. Severity of heart failure was assessed according to the Killip classification. In-hospital bleedings was defined using Thrombolysis in Myocardial Infarction (TIMI) criteria. TIMI major bleeding was defined as intracranial hemorrhage or a decrease in hemoglobin (Hb) levels from admission to discharge greater than or equal to 5 g/L. Minor bleeding was defined as a 3-5 g/dL decline in Hb, and minimal bleeding was defined as a less than 3 g/dL decline in Hb.

BMI was calculated using the ratio of body weight in kilograms and the square of the height in meters. Patients were classified into 6 groups according to age (the younger and elderly groups consisting of patients ≤65 and > 65 years old) and baseline body mass index BMI (normal weight, BMI < 24 kg/m^2^; overweight, 24 kg/m^2^ ≤BMI < 28 kg/m^2^; obese, BMI ≥ 28 kg/m^2^).

### 2.6. Statistical Analyses

Continuous variables were expressed as mean ± standard deviation (SD) unless otherwise indicated. Chi-square test was used to test for the association between categorical variables and one-way ANOVA for the association between categorical and continuous variables. Multivariate analysis was performed to analyze factors influencing prognosis in different age groups. All of the baseline clinical characteristics and treatment characteristics were included and analyzed to perform binary logistic regression. Treatment characteristics were medication received after discharge and lifestyle. Statistical analyses were performed using the SPSS for Windows statistical software package version 18.0 (SPSS Inc., Chicago, IL, USA). A 2-tailed *p* value <0.05 was considered statistically significant.

## 3. Results

**Figure 1:**
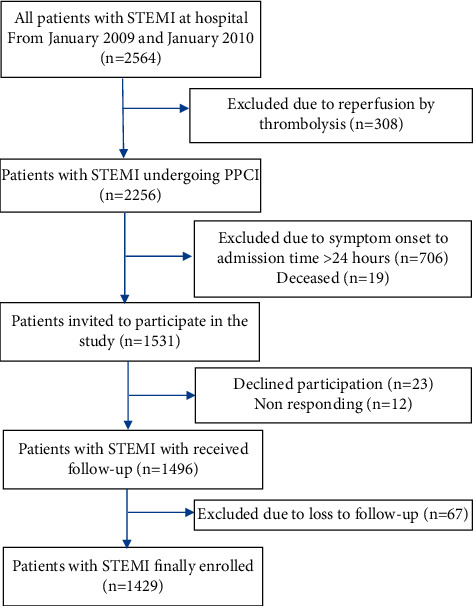
Flowchart of the study design. STEMI, ST-segment elevation myocardial infarction; PPCI, primary percutaneous coronary intervention.

In total, 2564 patients were hospitalized with STEMI in the Department of Cardiology in the medical institution in Beijing between January 2009 and January 2010. After excluding all participants that either did not meet the inclusion criteria or who could not be accessed, 1531 patients were eligible. Of those, 23 patients declined participation, 16 did not respond to the invitation, and 67 were lost to follow-up. Finally, a total of 1429 STEMI patients treated with PPCI were included in the present study (Figure 1). The mean age of participants was 58.0 ± 11.8 years, in which 83.1% were male, and the mean BMI was 26.4 ± 3.3 kg/m^2^. Among the patients in the younger age group, there were 168 patients with normal weight (16.9%), 596 patients with overweight (60.1%), and 228 patients with obesity (23.0%). Among the patients in the elderly age group, there were 50 patients with normal weight (11.4%), 198 patients with overweight (45.3%), and 189 patients with high BMI (43.2%).

### 3.1. Baseline Characteristics

Baseline characteristics of the patients sorted by age and BMI are shown in Table 1. In the younger age group, patients who were obese had a higher prevalence of diabetes and hypertension compared to those who had normal BMI and overweight patients. Obese patients also had higher admission blood pressure and triglyceride (TG) level and were more frequently on beta-blockers in comparison with overweight and normal-weight patients. Compared with overweight and obese patients, normal-weight patients had higher peak CK-MB. In the older age groups, obese patients were less frequently on beta-blockers before STEMI and had higher low-density lipoprotein cholesterol (LDL-C) and uric acid levels compared with their thinner counterparts. There were no differences in age, glucose, high-sensitivity C-reactive protein (hs-CRP), brain natriuretic peptide (BNP) level, stent length, and prevalence of left ventricular aneurysm among the three BMI groups for both age groups.

The incidence of bleeding was not significantly different among the three BMI groups in the younger age group, and the obesity group had a significantly lower incidence of bleeding in the older group.

### 3.2. Persistence with Therapy after Discharge and Lifestyle Characteristics

Persistence rates for aspirin, statins, beta-blockers, ACEI/ARBs, and lifestyle characteristics of the patients at 60 months after discharge are shown in Table 2. The frequency of aspirin and statin use was relatively lower in the normal-weight group than in the other groups in the younger age group. In contrast, the frequency of ACEI/ARB and statin use was significantly higher in the normal-weight group in comparison with overweight and obese patients in the elderly age group.

Compared with normal-weight patients, obese patients were less frequently on regular exercise in the younger age groups. Adherence to reduced fried food and meet was significantly higher in normal-weight patients than overweight and obesity patients in the older age group.

### 3.3. Clinical Outcomes

During the follow-up periods (median 59 months), 13.9% of patients experienced the adverse event rates. The primary outcome occurred in 15.5%, 14.2%, and 7.9% of normal-weight, overweight, and obese patients, respectively (*p* = 0.033) among the patients in the younger age group. In the elderly age group, the primary outcome was 12.0%, 10.0%, and 23.8% in normal-weight, overweight, and obese patients, respectively (*p* = 0.001) (Table 3).

### 3.4. Predictors of the Primary Outcome

Multivariate logistic regression analyses for the primary outcome are summarized in Table 4. In model 2, we adjusted for age, sex, SBP, DBP, HR, glucose, LDL-C, hypertension, diabetes, smoking, baseline TIMI flow grade, medication adherence, and bleeding. In the younger age group, low BMI was a significant predictor of the primary outcome. The odds ratios (OR) for overweight in comparison with normal weight was 0.741 (95% CI: 0.413–0.979; *p* = 0.038), the OR for obesity in comparison with normal-weight patients was 0.508 (95% CI: 0.344–0.750; *p* = 0.016). History of diabetes (OR = 4.286, [2.398, 7.660]; *p* < 0.001), adherence of regular exercise (OR = 0.519, [0.351, 0.768]; *p* < 0.001), and beta-blockers (OR = 0.508, [0.344, 0.750]; *p* < 0.001) were the other independent predictors of the primary outcome, while diabetes (OR = 2.630, [1.110, 6.234]; *p* = 0.028), hypertension (OR = 3.444, [1.429, 8.330];*p* = 0.006), triple disease (OR = 2.712, [1.290, 5.688]; *p* = 0.008), regular exercise (OR = 0.472, [0.234, 0.951]; *p* = 0.036), ACEI/ARB (OR = 0.298, [0.144, 0.618]; *p* = 0.001), and bleedings (OR = 12.670, [3.822, 42.001]; *p* < 0.01) were associated with the primary outcome in the elderly group.

### 3.5. Sensitivity and Specificity Analysis

For the younger age patients, area under the curve (AUC) for the ability of predictors including BMI, diabetes history, regular exercise, and usage of beta-blockers to predict primary endpoint was 0.652 (95% CI: 0.616–0.686; *p* < 0.01). The diagnostic parameters were as follows: sensitivity, 75.0%; specificity, 53.6%. For the elderly age group, when predictors (diabetes history, hypertension history, triple disease, regular exercise, usage of ACEI/ARB, and bleeding) were used in combination, AUC value was 0.662 (95% CI 0.626–0.697; *p* < 0.01), sensitivity was 85.1%, and specificity was 41.8%.

## 4. Discussion

Obesity is recognized as traditional risk factor in the development for coronary artery disease. Several chronic diseases demonstrated an obesity paradox where a higher BMI may be associated with lower mortality and with better outcome, including percutaneous intervention, AMI, and heart failure [15, 16].

In this study involving patients presenting with STEMI undergoing PPCI, there was no difference in long-term outcomes between normal-weight, overweight, and obese patients >65 years old. In the younger patients, however, low BMI was found to be a significant predictor of all-cause mortality, revascularization, and AMI on multivariate analysis. The results show that the obesity paradox was recognized only in the ≤65-year-old group.

In the field of cardiovascular medicine, an obesity paradox remains controversial. Many studies have reported an obesity paradox in AMI and post-PCI. For example, in a retrospective study which included 5338 patients with AMI and a mean follow-up of 2.8 years, obesity patients had lower mortality and recurrent AMI incidence than normal-weight individuals [17]. A large meta-analyses of AMI patients concluded that both in-hospital and long-term mortality rates were lower in the overweight versus the normal-weight group [18]. However, in an analysis of 2,238 patients undergoing PPCI for STEMI, BMI was not associated with 1-year rates of death [19]. In a prospective study of 478 STEMI and left ventricular dysfunction patients, normal-weight, overweight, and obese patients had similar in-hospital outcomes and 30-day outcomes [14].

The mechanisms for obesity paradox in AMI are unclear, but there are several potential theories. One explanation is that overweight or obese patients tend to be younger in some studies. Eugenia et al. reported that though obese patients with AMI have an improved prognosis after PPCI compared with normal-weight patients, BMI itself was not an independent predictor of survival; the greater survival in the obese patient with AMI after PPCI was attributable to the association of this condition with younger age [20]. In our study, we investigated the impact of age on obesity paradox in AMI patients who underwent PPCI. We separate our data into younger and elderly patients and age was not found to be significantly different among the three BMI groups for both age groups.

Some investigators have suggested that an unmeasured variable that confounded the association between BMI and the risk for adverse clinical outcomes may also explain the presence of the paradox. For example, the presence of chronic disease in the lower BMI groups might also explain the worse prognosis of these patients [21, 22]. However, in the present study, there was no significant difference in the prevalence of previous PCI and stroke across BMI categories. The obese patients had a higher prevalence of diabetes and hypertension compared to those who had normal weight and overweight patients in the younger group. In addition, smoking has also been suggested as a confounder in the relationship of obesity and prognosis. Some studies have suggested that smoking may be associated with lower BMI [23]. However, those were not the case in our study.

Several research studies suggested that the favorable results in patients with the higher BMI quartile may be related to the optimal management. Obese patients usually were treated more aggressively. Diercks et al. reported that patients with higher BMI quartiles were more frequently taking guideline-recommended therapies on admission including aspirin, beta-blockers, and statin [24]. In the present study, we revealed that obese patients were more frequently on beta-blockers before STEMI in the younger age group. Furthermore, the present study evaluated the long-term medication adherence of the three groups of patients. The result revealed that more obesity patients ≤65 years of age received aspirin and statin as compared with normal-weight and overweight patients in the 5 years after discharge. The optimal adherence of guideline-directed therapies in obesity patients may partly explain the obesity paradox in the younger group.

Our findings have important implications because we have adjusted multiple confounders. In the elderly group, patients with low BMI had similar adverse cardiac events compared to those in other BMI categories in this study. This is not consistent with some previous reports. For example, Fukuoka et al. reported that low BMI in the elderly age group was an independent predictor of all-cause mortality [25]. Previous reports showed that the obesity paradox may be explained by the fact that obese patients have less postprocedural complications, such as bleeding complications, compared to overweight or underweight patients [26, 27]. Antiplatelet drugs are rarely dose-adjusted for weight, and leaner patients usually have increased risk of bleeding. However, bleeding complications were not assessed in previous study in evaluating the associations between obesity and mortality. In the present study, in order to evaluate the influence of the confounding factor, we analyzed the bleeding complications. Bleeding complication was higher in normal-weight patients compared to overweight and obese patients: normal weight, 14.3; overweight, 7.8; obese, 3.5. After adjusted for bleeding complication, BMI was not an independent predictor of the primary outcome in the elderly group.

We also evaluated the lifestyles including physical activity and diet. Regular exercise could decrease the risk of death and recurrent AMI in both age groups. In the younger group, although the percentage of patients who exercise regularly was significantly lower in the obesity group, the low BMI patients had a higher incidence of adverse cardiac events. These findings indicate that low BMI in patients with STEMI is likely to be associated with poor prognosis for patients ≤65 years of age.

Our study had several limitations. First, the present study was a single-center study, which might have led to recruitment bias. Second, we used the BMI at the onset of STEMI, but it was not reevaluated during the follow-up, and it may have effects on the results. Third, the present study had a relatively limited sample size; thereby, the results should be validated through studies with a larger cohort.

## 5. Conclusion

The prognostic impact of BMI may differ by age in STEMI patients with primary PCI. Younger age patients with higher levels of baseline BMI had favorable clinical outcomes. However, the obesity paradox was not recognized in the elderly age group.

## Figures and Tables

**Table 1:** Baseline profile and treatment characteristics of younger and older STEMI patients.

	≤65 y			*p* value	>65 y			*p* value
	BMI				BMI			
	Normal	Overweight	Obesity		Normal	Overweight	Obesity	
	<24 (*n* = 168)	24–28 (*n* = 596)	>28 (*n* = 228)		<24 (*n* = 50)	24–28 (*n* = 198)	>28 (*n* = 189)	
Age, years	52 ± 9	52 ± 8	51 ± 8	0.847	71 ± 4	72 ± 4	72 ± 4	0.444
Male, *n* (%)	146 (86.9)	492 (82.6)	198 (86.8)	0.187	34 (68.0)	170 (85.9)	147 (77.8)	0.008
Hypertension, *n* (%)	84 (50.0)	304 (51.0)	150 (65.8)	<0.001	28 (56.0)	122 (61.6)	108 (57.1)	0.314
Diabetes mellitus, *n* (%)	44 (26.2)	124 (20.8)	78 (34.2)	<0.001	8 (16.0)	58 (29.3)	48 (25.4)	0.099
Smoking status, *n* (%)				0.042				<0.01
Current smoking	96 (57.1)	352 (59.1)	144 (63.2)		22 (44.0)	130 (65.7)	129 (68.3)	
Ex-smoking	4 (2.4)	28 (4.7)	12 (5.3)		4 (8.0)	4 (2.0)	12 (6.3)	
Never	68 (40.5)	216 (36.2)	72 (31.6)		24 (48.0)	64 (32.3)	48 (25.4)	
Previous stroke, *n* (%)	6 (3.6)	28 (4.7)	6 (2.6)	0.381	8 (14.3)	20 (10.0)	21 (11.1)	0.063
Previous PCI, *n* (%)	6 (3.6)	36 (6.0)	6 (2.6)	0.088	2 (3.6)	0 (0)	0 (0)	0.001
Medication before AMI, *n* (%)								
Aspirin	10 (6.0)	56 (9.4)	30 (13.2)	0.053	4 (8.0)	16 (8.1)	6 (3.2)	0.207
ACEIs/ARBs	18 (10.7)	68 (11.4)	36 (15.8)	0.182	8 (16.0)	36 (18.2)	24 (12.7)	0.627
Beta-blocker	10 (6.0)	56 (9.4)	36 (15.8)	0.003	12 (24.0)	16 (8.1)	6 (3.2)	<0.01
Statins	4 (2.4)	28 (4.7)	12 (5.3)	0.343	4 (8.0)	4 (2.0)	0 (0)	0.003
Time-to-hospital admission, h	13 ± 14	17 ± 26	19 ± 26	0.056	11 ± 14	20 ± 25	18 ± 27	0.330
Physical findings on admission								
SBP, mmHg	113 ± 26	121 ± 17	121 ± 22	<0.01	114 ± 27	118 ± 18	121 ± 18	0.551
DBP, mmHg	72 ± 14	76 ± 12	76 ± 15	0.005	75 ± 11	73 ± 12	74 ± 10	0.553
HR, beats/min	75 ± 16	76 ± 11	78 ± 19	0.223	76 ± 12	79 ± 18	78 ± 16	0.377
Glucose, mmol/L	5.4 ± 1.9	7.7 ± 2.9	5.4 ± 2.7	0.521	4.7 ± 1.9	5.1 ± 2.3	5.4 ± 1.6	0.291
hs-CRP, mmol/L	8.5 (0.7–42.6)	9.6 (0.2–42.5)	8.8 (0.2–41.2)	0.517	8.1 (0.6–34.3)	11.7 (0.9–40.9)	10.9 (0.22–43.3)	0.743
BNP, mmol/L	68.8 (2.4–1886)	121.7 (3.7–3217)	99.5 (4.9–2381)	0.415	5.8 (0.1–135)	264 (39.6–3321)	130 (2.7–2100)	0.258
LDL-C, mmol/L	2.8 ± 0.8	2.9 ± 0.8	3.0 ± 0.6	0.311	2.7 ± 0.6	2.9 ± 0.9	3.1 ± 0.7	0.048
TG, mmol/L	1.8 ± 1.0	2.0 ± 1.3	2.4 ± 1.6	0.002	1.9 ± 1.3	1.9 ± 1.5	2.1 ± 1.1	0.308
Uric acid, umol	300 ± 106	308 ± 95	307 ± 118	0.705	318 ± 115	296 ± 80	352 ± 110	0.005
Peak CK-MB, mU/ml	391 ± 995	210 ± 200	209 ± 223	0.004	218 ± 188	237 ± 239	298 ± 293	0.141
Killip classes ≥2	132 (78.6)	440 (73.8)	144 (63.2)	0.001	38 (76.0)	134 (67.7)	135 (71.4)	0.390
IRA, *n* (%)				0.012				0.308
LM	4 (2.4)	4 (0.7)	0 (0)		0 (0)	0 (0)	0 (0)	
LAD	86 (51.2)	296 (49.7)	138 (60.5)		30 (60.0)	104 (52.5)	102 (54)	
LCX	36 (21.4)	80 (13.4)	18 (7.9)		6 (12.0)	24 (12.1)	42 (22.2)	
RCA	42 (25.0)	216 (36.2)	72 (31.6)		14 (28.0)	68 (34.3)	45 (23.8)	
Triple disease, *n* (%)	38 (22.6)	152 (25.5)	66 (28.9)	0.351	18 (36.0)	52 (26.3)	30 (15.9)	0.048
Initial TIMI flow 0, *n* (%)	90 (53.6)	356 (59.7)	138 (60.5)	0.303	36 (72.0)	146 (73.7)	117 (61.9)	0.036
Stent length, mm	24 ± 6	24 ± 6	25 ± 7	0.452	24 ± 6	24 ± 7	25 ± 6	0.804
Stent diameter, mm	2.7 ± 0.9	2.8 ± 0.8	3 ± 0.7	0.015	2.9 ± 0.7	2.9 ± 0.6	2.9 ± 0.5	0.862
Echocardiography								
LVEF, %	55 ± 11	54 ± 10	54 ± 9	0.898	53 ± 12	56 ± 9	53 ± 7	0.085
LVESD, mm	33 ± 4	34 ± 7	35 ± 7	0.098	34 ± 4	31 ± 6	39 ± 9	0.004
LVEDD, mm	50 ± 21	48 ± 7	49 ± 6	0.343	48 ± 3	48 ± 5	49 ± 4	0.357
LVA, *n* (%)	6 (3.6)	20 (3.4)	12 (5.3)	0.435	4 (8.0)	12 (6.1)	18 (9.5)	0.235
Bleedings, *n* (%)	22 (13.1)	38 (6.4)	12 (5.3)	0.514	8 (16.0)	8 (4.0)	2 (1.1)	0.018
TIMI major bleeding	0	0	0		0	0	0	
TIMI minor bleeding	6 (3.6)	12 (2.0)	3 (1.3)		1 (2.0)	0	0	
TIMI minimal bleeding	16 (9.5)	26 (4.4)	9 (3.9)		7 (14.0)	8 (4.0)	2 (1.1)	

Values are *n* (%) or means ± SD. BMI, body mass index; PCI, percutaneous coronary intervention; AMI, acute myocardial infarction; ACEIs, angiotensin-converting enzyme inhibitors; ARBs, angiotensin receptor blockers; SBP, systolic blood pressure; DBP, diastolic blood pressure; HR, heart rate; hs-CRP, high-sensitivity C-reactive protein; BNP, B-type natriuretic peptide; LDL-C, low-density lipoprotein cholesterol; TG, triglyceride; CK, creatine kinase; IRA, infarct-related artery; LM, left main; LAD, left anterior descending artery; RCA, right coronary artery; LCX, left circumflex artery; TIMI, Thrombolysis in Myocardial Infarction grade; LVEF, left ventricular ejection fraction; LVEDD, left ventricular end-diastolic dimension; LVESD, left ventricular end-systolic diameter; LAV, left ventricular aneurysm.

**Table 2:** Persistence with therapy and lifestyle characteristics after discharge for younger and older STEMI groups.

	≤65 y			*p* value	>65 y			*p* value
	BMI				BMI			
	Normal	Overweight	Obesity		Normal	Overweight	Obesity	
	<24 (*n* = 168)	24–28 (*n* = 596)	>28 (*n* = 228)		<24 (*n* = 50)	24–28 (*n* = 198)	>28 (*n* = 189)	
Medication adherence								
Aspirin	118 (70.2)	488 (81.9)	198 (86.8)	<0.01	42 (84.0)	174 (87.9)	150 (79.4)	0.196
ACEI/ARB	32 (19)	120 (20.1)	48 (21.1)	0.660	18 (36.0)	38 (19.2)	48 (25.4)	0.035
Beta-blockers	68 (40.5)	228 (38.3)	96 (42.1)	0.298	20 (30.0)	92 (46.5)	66 (34.9)	0.284
Statin	96 (57.1)	400 (67.1)	156 (68.4)	<0.01	38 (76.0)	146 (73.3)	102 (54.0)	0.012
Lifestyle								
Smoking cessation	24 (14.3)	80 (13.4)	24 (10.5)	0.455	6 (10.7)	54 (27.3)	33 (17.5)	0.009
Regular exercise	112 (66.7)	332 (55.7)	114 (50.0)	0.020	34 (68.0)	120 (60.6)	96 (50.8)	0.885
Diet, reduced fried foods and meat	48 (28.6)	192 (32.2)	96 (42.1)	0.111	10 (20.0)	90 (45.5)	48 (25.4)	0.002
Sleeping time	6.7 ± 1.3	6.9 ± 1.2	6.8 ± 1.1	0.022	7.2 ± 1.1	7.2 ± 1.4	7.1 ± 1.2	0.702

BMI, body mass index; ACEIs, angiotensin-converting enzyme inhibitors; ARBs, angiotensin receptor blockers.

**Table 3:** Clinical outcomes according to body mass index category.

	≤65 y			*p* value	>65 y			*p* value
	BMI				BMI			
	Normal	Overweight	Obesity		Normal	Overweight	Obesity	
	<24 (*n* = 168)	24–28 (*n* = 596)	>28 (*n* = 228)		<24 (*n* = 50)	24–28 (*n* = 198)	>28 (*n* = 189)	
Primary endpoint	26 (15.5%)	84 (14.1%)	18 (7.9%)	0.033	6 (12.0%)	20 (10.1%)	45 (23.8%)	0.001
Death	6 (3.6%)	28 (4.7%)	0 (0)	0.004	4 (8.0%)	12 (6.1%)	3 (1.6%)	0.039
Revascularization	18 (10.7)	32 (5.4%)	18 (7.9%)	0.041	0 (0)	4 (2.0%)	24 (12.7%)	<0.001
AMI	18 (10.7%)	44 (7.4%)	18 (7.9%)	0.209	2 (4.0%)	4 (2.0%)	24 (12.7%)	<0.001

BMI, body mass index; AMI, acute myocardial infarction.

**Table 4:** Multivariate analysis for major adverse cardiac events according to age.

	≤65 years			
	Model 1 ^†^OR (95% CI)	*p* value	Model 2 ^‡^OR (95% CI)	*p* value
BMI				
<24	1		1	
24–28	0.436 (0.175–0.927)	0.001	0.741 (0.413–0.979)	0.038
>28	0.360 (0.203–0.636)	<0.001	0.508 (0.344–0.750)	0.016
Diabetes history	5.013 (2.730–8.130)	<0.001	4.286 (2.398–7.660)	<0.001
Lifestyle and medication after discharge				
Regular exercise	0.562 (0.382–0.827)	0.003	0.519 (0.351–0.768)	0.002
Beta-blockers	0.524 (0.357–0.770)	0.001	0.508 (0.344–0.750)	0.001

	>65 years			
	Model 1 ^†^OR (95% CI)	*p* value	Model 2 ^‡^OR (95% CI)	*p* value
Diabetes history	2.701 (1.137–6.452)	0.026	2.630 (1.110–6.234)	0.028
Hypertension	3.735 (1.156–9.858)	0.001	3.444 (1.429–8.300)	0.006
Triple disease	2.701 (1.217–5.694)	0.001	2.712 (1.290–5.688)	0.008
Lifestyle and medication after discharge				
Regular exercise	0.419 (0.212–0.826)	<0.001	0.472 (0.234–0.951)	0.036
ACEI/ARB	0.394 (0.203–0.763)	<0.001	0.298 (0.144–0.618)	0.001
Bleedings	12.980 (3.879–43.027)	<0.001	12.670 (3.822–42.001)	<0.001

BMI, body mass index; ACEIs, angiotensin-converting enzyme inhibitors; ARBs, angiotensin receptor blockers. ^†^Nonadjusted model. ^‡^The multivariable models were adjusted for the following covariate set: age, sex, SBP, DBP, HR, glucose, LDL-C, hypertension, diabetes, stroke, smoking, baseline TIMI flow grade, medication adherence, and bleeding.

## Data Availability

The data that support the findings of this study are available from the corresponding author upon reasonable request.
